# Who does not participate in telehealth trials and why? A cross-sectional survey

**DOI:** 10.1186/s13063-015-0773-3

**Published:** 2015-06-05

**Authors:** Alexis Foster, Kimberley A Horspool, Louisa Edwards, Clare L Thomas, Chris Salisbury, Alan A Montgomery, Alicia O’Cathain

**Affiliations:** School of Health and Related Research, University of Sheffield, Regent Court, 30 Regent Street, Sheffield, S1 4DA UK; Centre for Academic Primary Care, NIHR School for Primary Care Research, School of Social and Community Medicine, University of Bristol, Canynge Hall, 39 Whatley Road, Bristol, BS8 2PS UK; Nottingham Clinical Trials Unit, University of Nottingham, C Floor, South Block, Queen’s Medical Centre, Nottingham, NG7 2UH UK

**Keywords:** Telehealth, Trials, Declines, Recruitment, Non-participation, Refusers

## Abstract

**Background:**

Telehealth interventions use information and communication technology to provide clinical support. Some randomised controlled trials of telehealth report high patient decline rates. A large study was undertaken to determine which patients decline to participate in telehealth trials and their reasons for doing so.

**Methods:**

Two linked randomised controlled trials were undertaken, one for patients with depression and one for patients with raised cardiovascular disease risk (the Healthlines Study). The trials compared usual care with additional support delivered by the telephone and internet. Patients were recruited via their general practice and could return a form about why they were not participating.

**Results:**

Of the patients invited, 82.9 % (20,021/24,152) did not accept the study invite, either by returning a decline form (n = 7134) or by not responding (n = 12,887). In both trials patients registered at deprived general practices were less likely to accept the study invite. Decline forms were received from 29.5 % (7134/24,152) of patients invited. There were four frequently reported types of reasons for declining. The most common was telehealth-related: 54.7 % (3889/,7115) of decliners said they did not have access or the skills to use the internet and/or computers. This was more prevalent amongst older patients and patients registered at deprived general practices. The second was health need-related: 40.1 % (n = 2852) of decliners reported that they did not need additional support for their health condition. The third was related to life circumstances: 27.2 % (n = 1932) of decliners reported being too busy. The fourth was research-related: 15.3 % (n = 1092) of decliners were not interested in the research.

**Conclusions:**

A large proportion of patients declining participation in these telehealth trials did so because they were unable to engage with telehealth or did not perceive a need for it. This has implications for engagement with telehealth in routine practice, as well as for trials, with a need to offer technological support to increase patients’ engagement with telehealth. More generally, triallists should assess why people decline to participate in their studies.

**Trial registration:**

The Healthlines Study has the following trial registrations: depression trial: ISRCTN14172341 (registered 26 June 2012) and CVD risk trial: ISRCTN27508731 (registered 05 July 2012).

## Background

“*Telehealth is the remote exchange of data between a patient at home and their clinician(s) to assist in diagnosis and monitoring typically used to support patients with Long Term Conditions*” [1]. Examples of telehealth include online cognitive behavioural therapy, home monitoring of health parameters such as blood pressure and internet-based health forums. Globally, telehealth is becoming a prominent part of healthcare delivery. Telehealth interventions are increasingly being tested in randomised controlled trials (RCTs). Some of these telehealth trials have reported high decline rates of over 75 % amongst potential participants [2, 3]. A recent review of 37 telehealth studies found an average decline rate of 32 %, but this varied between 4 % and 71 % for the individual studies; and only two studies were RCTs [4]. Decline rates may vary because of the health condition under study. For example, some patient groups are known to be especially difficult to recruit, such as those with depression [5]. An alternative explanation is that selection criteria may have differed between trials. It is also possible that the nature of the telehealth interventions being tested affected the decline rates due to varying levels of acceptability amongst potential participants.

High decline rates in RCTs generally are problematic because they may result in underpowered trials or extended recruitment periods which have resource implications [6]. Additionally, if those declining are not representative of the clinical population, external validity may be compromised [7]. High decline rates may also indicate problems with the acceptability of the intervention, with implications for its uptake when delivered in routine practice. This latter issue may be particularly relevant to telehealth trials, since there may be physical barriers in terms of accessing technology [8], as well as psychological barriers such as low confidence in using the technology [9]. Understanding which types of patients do not participate in telehealth trials, and why, may help to improve recruitment rates, external validity and interpretation of trial results.

There has been some research examining the types of patients who do not participate in telehealth trials. No gender differences have been found in telehealth trials [2] or telehealth studies which use other methodologies [4]. Older adults are more likely not to participate in telehealth trials [2, 10] due to technological demands and because they have less access to technology [8, 9, 11]. This higher non-participation rate amongst older people also mirrors RCTs more generally [12, 13]. In terms of differences in participation amongst socio-economic groups, one telehealth trial did not find any differences [2]. However, studies of routine telehealth services report lower uptake amongst patients from lower socio-economic groups [14]. This mirrors the evidence based more generally on healthcare research, where there are lower response rates from patients experiencing greater socio-economic deprivation [15, 16]. There is no evidence on participation in telehealth trials by ethnic minority groups. However, these groups are usually under-represented in RCTs in the United Kingdom and North America, partly because a common exclusion criterion is the inability to speak English fluently [17].

To date, only a handful of studies have explored why patients do not participate in telehealth studies [2, 3, 8, 18]. These have been fairly small quantitative studies of 625, 331 and 79 patients [18, 2, 3], with only one qualitative study with 19 patients [8]. There was a review of why patients may not participate in telehealth research, but this was in relation to telehealth studies in general, rather than RCTs specifically [4]. Eight main reasons for declining participation in telehealth trials have been identified. First, patients have concerns, and in some cases anxiety, regarding the technological aspects of the intervention [2, 8, 18]. Second, some patients do not perceive the intervention to be beneficial [3, 18]. Third, some patients prefer face-to-face care, rather than healthcare delivered remotely, which is an integral component of telehealth interventions [2]. Fourth, some patients believe that there is no need for the intervention, for example, because routine healthcare is sufficient [3]. Fifth, many patients decline due to poor health [2, 19] or perceive their older age to be a barrier [2]. Sixth, patients are simply not interested [4], but it is not clear whether patients are not interested in the telehealth intervention or the research itself. Seventh, patients simply do not want to participate in the study [18]. Finally, some patients are too busy to participate [18]. These mirror some of the reasons why patients decline to participate in RCTs more generally: patients are not interested or are too busy, are too ill, perceive themselves as ineligible, have transport barriers, have concerns about the intervention or do not want to be randomised [20–22].

Given the high decline rate in some telehealth trials and the limited literature investigating this topic, it is important to understand more about which patients do not choose to participate in telehealth trials and their reasons for this choice. We utilised recruitment data for two large linked RCTs of telehealth (the Healthlines Study) to undertake a quantitative study of who does not participate in telehealth trials and why.

## Methods

### The Healthlines Study

The Healthlines Study consists of two linked RCTs of a telehealth intervention, one for patients with depression and one for patients with a raised risk of cardiovascular disease (CVD risk) [23]. The telehealth intervention consisted of up to 12 months of telephone support from a health information advisor and access to a range of online resources, including computerised cognitive behavioural therapy software packages, support for home monitoring (such as blood pressure monitoring) and links to online educational and support tools. To participate in the trials, patients had to have at least weekly access to an email account and the internet. The trials were approved by the National Research Ethics Service Committee South West-Frenchay (Reference 12/SW/0009) and have the following trial registrations: depression trial: ISRCTN14172341 (registered 26 June 2012) and CVD risk trial: ISRCTN27508731(registered 05 July 2012).

### Recruitment of patients

Patients were recruited via 43 general practices between June 2012 and July 2013. Searches of practice records were undertaken to identify potential participants. For the depression trial, patients were identified who were aged 18 to 100 years old, and in the preceding three months to the search visit were coded as having depression, anxiety or low mood, or who were issued an antidepressant prescription. Exclusion criteria were applied such as having psychosis, substance abuse issues or dementia. For the CVD risk trial, patients were selected who were aged 40 to 74 years and, based on data in routine general practice records, were estimated to have at least a 19 % risk of having a cardiovascular event in the next ten years and had one or more modifiable risk factors (being a smoker, overweight or having high blood pressure). Exclusion criteria were applied including being identified as part of the depression trial or having had a cardiovascular event, such as a heart attack or stroke. General practitioners at each practice were asked to exclude any patients they felt were unsuitable, for example, the recently bereaved [23]. After exclusions, 16,570 patients were invited to participate in the depression trial, and 7582 patients in the CVD risk trial. A larger number of patients were invited in the depression trial because it was anticipated that there would be a lower participation rate.

Practice staff sent the selected patients an information pack, including a personalised invitation letter, a patient information booklet, an acceptance form, a decline form and a freepost envelope. Approximately three weeks later, patients who had not responded were re-sent the information packs.

### Acceptors, active decliners and non-responders

Invited patients could return an acceptance form (acceptors). On receipt of this form, they were contacted by a researcher to assess their eligibility. Alternatively, patients could return a decline form (active decliners), or choose not to respond (non-responders). The decline form was anonymous, but linked to demographic information from the general practices through a study ID number. Patients were asked to indicate why they were declining to participate by ticking one or more of the pre-specified reasons provided on the form, including:

- I do not have regular access to the internet or an email address
- I do not feel confident enough with computers
- I am too busy at the moment
- I do not feel I need any more support with my health at this time
- I am not interested in this research

The pre-specified reasons incorporated the common reasons for declining reported in previous literature on uptake in telehealth studies and RCTs more generally. In addition, there was an ‘other reason’ that responders could select, and they were asked to specify this reason in a free text box. A small proportion of patients (4.2 %, n = 1020/24,152) were sent a decline form with the additional pre-specified reason of ‘I do not understand the study’. This was part of a sub-study where patients were sent a re-designed written patient information sheet to explore whether this increased recruitment [23].

Data on the age, sex and ethnicity of invited patients was collected from the general practice records. However, ethnicity was poorly recorded in practice records, and the study had to reply on self-report from patients who returned forms. For this reason, ethnicity is missing for non-responders. Levels of socio-economic deprivation were derived from the general practice at which patients were registered.

### Analysis

A two-stage analysis was conducted. The first stage was a comparison of the socio-demographic characteristics of the different response types. This included comparing the acceptors with a combined category of active decliners and non-responders. A comparison of active decliners and non-responders was then conducted to understand the effect of non-response bias on our study of why patients declined. Data were entered into SPSS. Ethnicity was re-categorised from multiple ethnicity categories into a dichotomous variable of White or Black and Minority Ethnicity (BME). A variable was created to capture socio-economic deprivation based on the general practice at which a patient was registered. Deprivation was defined by the decile attributed to a general practice in the National Practice Profiles [24]. Patients registered at practices in deciles 1–5 were coded as ‘deprived’ and patients registered at practices in deciles 6–10 were coded as ‘affluent’. Chi-square and independent *t*-tests were undertaken to test for differences by age, gender, ethnicity and deprivation.

The second stage involved exploring the reasons why patients declined to participate in the trials. Frequencies and percentages were calculated to understand the prevalence of each reason. The other reasons patients provided for declining were coded by one researcher (AF) and checked by another researcher (KAH). Of the 979 patient-reported ‘other’ comments in the depression trial, the researchers initially disagreed on the coding for 204 (20.8 %). Of the 551 CVD risk ‘other’ comments, there was disagreement on 63 (11.4 %). These comments were discussed and consensus reached on coding.

As a considerable proportion of responders declined because of technology-related reasons, and this issue is intrinsic to telehealth, further analysis was conducted. Responders were categorised as declining for a technology-related reason if they had selected the pre-specified reasons of no internet access or no computer confidence, or if they provided their own reason related to technology issues, such as not having a computer. Technology-related decliners were compared with other types of decliners by age, gender, ethnicity and deprivation. Chi-square and independent *t*-tests were undertaken.

## Results

### Responses to the Healthlines Study invitation

Overall, 82.9 % (20,021/24,152) of the patients invited did not accept the study invite (Table 1). Most of these patients (n = 12,887) did not respond at all, but decline forms were received from 29.5 % (n = 7134) of all patients invited (Table 1). Response type differed by health condition, with patients with depression more likely not to accept than those with raised CVD risk (84 % depression versus 80 % CVD risk, *P* ≤ 0.001, *X*^2^ = 452.8, df = 1).

**Table 1:** Frequency of response type by health condition

	Type of response			
	Accepted	Declined	Non-responders	Total
	N %	N %	N %	N %
Depression RCT	2581 (15.6)	4382 (26.4)	9607 (58.0)	16,570 (100)
CVD RCT	1550 (20.4)	2752 (36.3)	3280 (43.3)	7582 (100)
Total	4131 (17.1)	7134 (29.5)	12,887 (53.4)	24,152 (100)

### Socio-demographic characteristics

#### Decliners (active decliners and non-responders) compared to acceptors

All those that declined the invite (active decliners and non-responders) were compared with those accepting the invite (Tables 2 and 3). In the depression trial, males (86 %, n = 4570) were more likely to decline than females (84 %, n = 9419), *P* ≤ 0.001, *X*^2^ = 11.43, df = 1. In the CVD risk trial, females (83 %, n = 1675) were more likely to decline than males (78 %, n = 4357 males), *P* ≤ 0.001, *X*^2^ = 18.26, df = 1. Although the differences were statistically significant, they were not large for the depression trial. Of those whose ethnicity was known, in the depression trial there was no difference in decline rates between white patients (87 %, n = 4152) and BME patients 92 %, n = 82), *P* = 0.168, *X*^2^ = 1.90, df = 1), although numbers were small. In contrast, there was some evidence in the CVD risk trial that patients from BME groups (82 %, n = 78) were more likely to decline than white patients (70 %, n = 2553), *P* = 0.08, *X*^2^ = 6.94, df = 1). Patients from deprived general practices were more likely to decline compared with patients from affluent general practices in both the depression and CVD risk trials (Depression: 87 %, n = 3947 deprived versus 84 %, n = 10,042 affluent declined, *P* ≤ 0.001, *X*^2^ = 21.4, df = 1; CVD risk: 85 %, n = 1725 deprived versus 78 %, n = 4307 affluent declined, *P* ≤ 0.001, *X*^2^ = 42.93, df = 1). In the depression trial, there was no difference in the mean age of decliners compared to acceptors (Mean age: Decliners: 50.66 years old versus Acceptors: 50.79, *P =* 0.710, *t* = −0.371). In the CVD risk trial, however, there was a statistically significant difference, with decliners being younger than acceptors (Mean age: Decliners: 65.75 years versus Acceptors: 66.33 years*, P* = 0.001, *t* = −3.321), although the mean difference in age was less than a year.

**Table 2:** The characteristics of the different response types for the depression trial

		Accepted n (%)	Declined n (%)	Non-responders n (%)	All N =100 %
Gender	Female	1825 (16.2)	3049 (27.1)	6370 (56.7)	11,244
	Male	756 (14.2)	1333 (25.0)	3237 (60.8)	5326
Ethnicity	White	609 (12.8)	4152 (87.2)	N/A	4761
	BME	7 (7.9)	82 (92.1)		89
Deprivation	Affluent	1967 (16.4)	3239 (27.0)	6803 (56.6)	12,009
	Deprived	614 (13.5)	1143 (25.1)	2804 (61.5)	4561
Age (mean, (SD))		50.8 (13.87)	59.7 (15.94)	46.5 (15.85)	50.7
Total		2581 (15.6)	4382 (26.4)	9607 (58.0)	N = 16,570

**Table 3:** The characteristics of the different response types for the CVD risk trial

		Accepted n (%)	Declined n (%)	Non-responders n (%)	All N =100 %
Gender	Female	347 (17.2)	907 (44.9)	768 (38.0)	2022
	Male	1203 (21.6)	1845 (33.2)	2512 (45.2)	5560
Ethnicity^a^	White	1118 (30.5)	2553 (69.5)	N/A	3671
	BME	17 (17.9)	78 (82.1)	N/A	95
Deprivation	Affluent	1235 (22.3)	2001 (36.1)	2306 (41.6)	5542
	Deprived	315 (15.4)	751 (36.8)	974 (47.7)	2040
Age (mean, (SD))		66.3 (5.59)	67.4 (5.24)	64.4 (6.2)	65.87
Total		1550 (20.4)	2752 (36.3)	3280 (43.3)	N = 7582

^a^Data on ethnicity was not available for non-responders

#### Active decliners compared to non-responders

Of the patients who did not accept the study invite, decline forms were received from 35.6 % (7134/20,021). The active decliners were compared to non-responders to understand how representative active decliners were of all patients who did not accept the study invite. In both the depression and CVD risk trials, females were more likely to return decline forms compared to males (Depression: 41 %, n = 3049 females versus 29 %, n = 1333 males, *P* ≤ 0.001, *X*^2^ = 14.67, df = 1; CVD risk: 54 %, n = 907 females versus 42 %, n = 1845 males, *P* ≤ 0.001, *X*^2^ = 67.95, df = 1). Ethnicity could not be compared because the data were not available for non-responders. In both trials, patients registered at affluent practices were more likely to return decline forms compared to patients registered at deprived practices (Depression: 32 %, n = 3239 affluent versus 29 %, n = 1143 deprived, *P* ≤ 0.001, *X*^2^ = 14.31, df = 1; CVD risk: 46 %, n = 2001 affluent versus 43 %, n = 751 deprived, *P* = 0.039, *X*^2^ = 4.24, df = 1), although differences were not large. In both trials, patients who returned decline forms were more likely to be older than non-responders (Depression: Mean age: Active decliners: 59.7 years versus Non-responders: 46.5 years, *P* ≤ 0.001, *t* = −45.679; CVD risk: Mean age: Active decliners: 67.4 years versus Non-responders: 64.4 years, *P* ≤ 0.001, *t* = −18.740).

### Reasons for declining

#### Number of reasons for declining

Of the 7134 decline forms received, 19 were blank. These have been included in the response type calculations above, but they have been excluded from the reasons for decline analysis below. Ninety-nine percent (n = 7045) of patients who returned a decline form provided a reason for declining to participate in the trial (Depression: 98.9 %, n = 4327; CVD risk: 99.1 %, n = 2718). Most decliners provided only one (Depression: 47.2 %, n = 2062; CVD risk: 39.4 %, n = 1079) or two reasons (Depression: 29.4 %, n = 1287; CVD risk: 33.6 %, n = 922).

In both health conditions, over 90 % of decliners selected at least one of the pre-specified reasons on the form (Depression: 90.4 %, n = 3954; CVD risk: 93.8 %, n = 2571). Some patients provided another reason, with more patients in the depression trial doing this compared to the CVD risk trial (Depression: 23.5 %, n = 1021; CVD risk: 16.1 %, n = 441). In both health conditions, a small number of responders specified a reason that was compatible with one of the pre-specified reasons (Depression: 62; CVD risk: 29). These were added to the frequencies of the pre-specified reasons. A small number of responders ticked the other reason option, but instead of specifying a reason, they wrote a comment, such as “thank you, but no thank you” (Depression: 17; CVD risk: 8); these are not included below.

People mainly ticked the pre-specified categories (Table 4), with one in five giving other reasons for declining (Table 5). Both the pre-specified reasons and the other reasons were categorised into four domains. These domains were developed thematically based on conceptual similarities.

**Table 4:** Pre-specified reasons for declining

Reason	Depression n (%)	CVD risk n (%)	Both conditions n (%)
No internet access	1834 (41.9)	1491 (54.4)	3325 (46.7)
No need for additional support with health issues	1717 (39.3)	1135 (41.4)	2852 (40.1)
No computer confidence	1580 (36.1)	1225 (44.7)	2805 (39.4)
Too busy	1214 (27.8)	718 (26.2)	1932 (27.2)
Not interested	648 (14.8)	444 (16.2)	1092 (15.3)
Do not understand what the research entails^a^	11/215 (5.1)	5/91 (5.5)	16/306 (5.2)
Other reason	1021 (23.5)	441 (16.1)	1462 (20.5)
Total N = 100 %	4377	2741	7118

^a^This option was only on the decline forms for 1020 patients as part of a sub-study. The 5.2 % is based on 306 decliners having this option on the form they returned

**Table 5:** Other reasons for declining

Category of reason	Reason	Depression n (%)	CVD risk n (%)	Both conditions n (%)
Health need-related	Health issues	195 (4.5)	50 (1.8)	245 (3.4)
	Satisfied with current support for their health	157 (3.6)	65 (2.4)	222 (3.1)
	Dissatisfied with health services	21 (0.5)	16 (0.6)	37 (0.5)
Patients’ lives	No space in their lives to participate in a trial	158 (3.6)	85 (3.1)	243 (3.4)
	Inconvenient at this time	104 (2.3)	85 (3.1)	189 (2.7)
Research-related	Perceive themselves as unsuitable	120 (2.7)	14 (0.5)	134 (1.9)
	Participation could cause distress	104 (2.4)	29 (1.1)	133 (1.9)
	Issues with the research	68 (1.6)	43 (1.6)	111 (1.6)
Telehealth-related	Dislikes the proposed intervention	77 (1.8)	25 (0.9)	102 (1.4)
	Practical barriers to participating	47 (1.1)	47 (1.7)	94 (1.3)
	Patient has communication difficulties	63 (1.4)	29 (11)	92 (1.3)
Unknown	Unknown	9 (0.2)	3 (0.1)	12 (0.2)
Total N = 100 %		4327	2718	7045

#### Telehealth-related reasons

Telehealth-related reasons, in terms of no computer confidence and/or no internet access, were prominent pre-specified reasons (Table 4), affecting 54.7 %, n = 3889 of active decliners. A small number of active decliners also specified that they were not participating because they did not like the telehealth intervention (1.4 %, n = 102), primarily because it was being delivered remotely rather than face-to-face; because of communication difficulties, such as a visual impairment, which meant that engagement in a telehealth intervention was problematic (1.3 %, n = 92); and because of practical barriers, such as not having a computer (1.3 %, n = 94).

#### Health need

A lack of perceived health need was the second most frequent option in the pre-specified reasons (40.1 %, n = 2852). A small number of people also declined because they were satisfied with their current level of support, such as having regular health checks (3.1 %, n = 222), or they were currently receiving treatment for other health conditions (3.4 %, n = 245).

#### Patients’ lives

The fourth most common reason for declining was being too busy (27.2 %, n = 1932). Some decliners provided other related reasons such as not having space in their life to participate in a trial, which sometimes related to not having the emotional capacity to participate (3.4 %, n = 243); or indicating that it was an inconvenient time to join the study, for example, because of planned time away from home (2.7 %, n = 189).

#### Research-related reasons

A lack of interest in the research was given as the fifth most common reason for declining (15.3 %, n = 1092). Related reasons were that patients perceived themselves as unsuitable for the trial, for example, because they did not have depression (1.9 %, n = 134); or the trial could be distressing, such as having to discuss health issues (1.9 %, n = 132). Both these reasons were more prevalent in the depression than in the CVD risk trial. A small number of patients (1.6 %, n = 111) specified other reasons related to the research, including confidentiality concerns and perceiving the research as a waste of money or resources.

### Technology-related reasons

Further analysis was conducted to compare responders who actively declined for technology-related reasons to those who actively declined for non-technology issues (Table 6). Decliners in the CVD risk trial were more likely to offer a technology-related reason than those in the depression trial (*P* ≤ 0.001, *X*^2^ = 126.93, df = 1). In the CVD risk trial, 63 % (n = 1729) of patients gave at least one reason for declining that was related to technology issues compared with 49 % (n = 2160) in the depression trial. There were some differences in the demographics of patients who declined for technology reasons compared with non-technology reasons. In the CVD risk trial, females were more likely to decline for a technology reason than males (*P* = 0.011, *X*^2^ = 6.52, df = 1), although there was no such difference in the depression trial (*P* = 0.508, *X*^2^ = 0.44, df = 1). Across both trials, there was no statistically significant difference in the ethnicity of patients who declined because of technology issues and those that did not (Depression: *P* = 0.590, *X*^2^ = 0.29, df = 1; CVD risk: *P* = 0.281, *X*^2^ = 1.16, df = 1), although the direction was that technology issues affected more people from BME communities. In both trials, patients were more likely to decline because of technology reasons if they were registered at deprived practices (Depression: *P* ≤ 0.001, *X*^2^ = 30.98, df = 1; CVD risk: *P* ≤ 0.001, *X*^2^ = 21.22, df = 1), or older (Depression: *P* ≤ 0.001, *t* = −35.683; CVD risk: *P* = 0.001, *t* = −3.352). This age difference was more pronounced in the depression trial, where there was a mean age difference of 15 years between patients who declined for technology reasons and those who declined for non-technology reasons compared with a one-year difference for CVD risk. This may be because there was a wider age range of patients included in the depression trial compared to the CVD risk trial.

**Table 6:** The characteristics of patients declining due to technology issues

		Depression			CVD risk		
Characteristic	Categories	Technology issues N (%)	Non-technology issues N (%)	Total N =100 %	Technology issues N (%)	Non-technology issues N (%)	Total N =100 %
Gender	Female	1493 (49)	1550 (51)	3043	601 (66)	304 (34)	905
	Male	667 (50)	663 (50)	1330	1128 (61)	709 (39)	1837
Ethnicity	White	2054 (49)	2101 (51)	4155	1615 (63)	938 (37)	2553
	BME	43 (52)	39 (48)	82	54 (69)	24 (31)	78
Deprivation	Deprived	625 (56)	483 (44)	1108	617 (69)	275 (31)	892
	Affluent	1510 (47)	1722 (53)	3232	1112 (60)	738 (40)	1,850
Age (Mean (SD))		67.4 (13.5)	52.3 (14.53)		67.6 (5.03)	66.9 (5.55)	

## Discussion

Although non-participation in telehealth trials is a widespread problem and may introduce bias, this issue has only been explored within small-scale studies. Our study appears to be the largest to date to examine two key issues related to this problem in patients with long-term conditions: the characteristics of patients who do not participate in telehealth trials (comparing over 24,000 invited patients) and the main reasons for not participating (responses from over 7000 patients). In total, 82.9 % of patients did not accept the study invite, which is comparable to other telehealth trials [2, 3]. Patients from deprived general practices were less likely to accept the study invite than those from affluent general practices. This contrasts with one telehealth trial which found no difference [2], but it is similar to findings in other health research generally [15, 16]. In the CVD risk trial, younger patients and BME patients were less likely to accept the study invite. However, the differences were small.

Overall, reasons for declining could be grouped into those that were specific to the telehealth intervention, those that were health need-related, issues related to patients’ lives and research-related reasons. Firstly, a large proportion of patients cited issues specific to telehealth interventions. They did not have access to a computer or the internet, or sufficient skill to use them; this has been found elsewhere [2, 4, 18]. The proportion of patients in this study declining because of technology reasons was high compared with a recent survey on telehealth, which reported that 68.5 % had computer technology available [9]. Our study and this latter survey were both undertaken as part of the Healthlines Study, and both included patients with depression and raised CVD risk. It may be that people in our study gave this answer as an easy option to decline or that they felt they needed more access to, or confidence with, technology in order to engage in these telehealth trials. It is also surprisingly high because in the patient information booklets for the Healthlines trials, it was explained to potential participants that methods were in place to facilitate access. For example, researchers could help patients to set up email accounts, and patients were allowed to use a family member or friend’s email address or computer.

Older patients were more likely to decline because of technology reasons, and this is consistent with previous research [11]. For example, internet availability rates have been reported as only 26.5 % amongst patients over 74 years old [9]. Consequently, telehealth may be more accessible for health conditions where patients are generally younger, for example, cystic fibrosis. Patients registered at general practices categorised as deprived were more likely to decline because of technology reasons, as found in research regarding the uptake of telehealth in general [14].

Secondly, a common reason for declining was patients not feeling a need for additional support with their health, reflecting previous literature [3]. Health problems could also create a barrier, reflecting an obstacle to participation in trials in general [2, 19]. However, this result does contrast with other studies about the acceptability of telehealth, which found that poor health was not related to less interest in telehealth [9]. Consequently, it may be that declining the trial due to health issues is related to participation in the trial rather than receiving the intervention.

Finally, there were a number of reasons offered that were generic to trials, not just telehealth trials [4, 18, 21, 25, 26]. Some were related to patients’ lives, such as being too busy. We identified an issue from the free text answers around emotional capacity to participate in terms of not having ‘space in their life’, for example, having caring responsibilities. A proportion of patients declined for geographical reasons, for example, because they were moving house or they were frequently away from home (for example, having a second home abroad). These are interesting both in the context of trials and routine practice of telehealth because they challenge a key principle of telehealth, which is that it is suitable for patients who have difficulty attending a general practice in person [27]. There were also some reasons given that were research-related, such as not being interested in participating. It was unclear whether this affected patients’ desire to participate in the trial or to receive the telehealth intervention. A small number of patients declined because they had issues with the research procedures, such as not wanting to be randomised, confidentiality concerns or regarding the study as a waste of money. These reasons are consistent with previous literature [25] and need to be better addressed in patient information booklets. However, such reasons were only a small proportion of the reasons that patients declined.

### Strengths and limitations

The key strength of the study is that it explored who does not participate in telehealth trials and the reasons why. It appears to be by far the biggest study exploring non-participation in telehealth trials, as the largest study we have identified included only 625 decliners. It also explores acceptability of telehealth trials in relation to two diverse long-term conditions, which is important as telehealth is often aimed at supporting patients with their long-term conditions [28].

There were some limitations. First, decline forms were only returned from 35.6 % of patients who did not accept the study invite. We do not know why patients did not respond. We have shown that active decliners were different from those who simply did not respond. These demographic differences may have an impact on the prevalence of the various reasons given for declining. For example, older people were more likely to return decline forms, but also to cite technology issues as the reason for declining. Therefore, technology reasons may be less of an issue in the wider patient population than in our sample. Second, we know little about the impact of ethnicity. The ethnicity of non-responders was not known in our study and there were only a small number of patients from BME groups in the decline population. Third, it was sometimes unclear whether patients were declining the trial or the telehealth intervention. Fourth, over 90 % of responders selected at least one of the pre-specified reasons for declining. This may be because they identified the key reasons why patients choose not to participate in a telehealth trial. However, there is the risk that patients may have selected those reasons because they were easy to tick. It is recommended that some of the other key reasons cited, such as health issues, be included in future decline forms. Fifth, the sample size was large and sometimes statistically significant differences were very small differences and therefore unlikely to be important. Sixth, this analysis is based on participation in two trials and the findings may be specific to the health conditions and the selection criteria for inviting people.

### Implications for telehealth trials and routine practice

The main implication of this research for telehealth trials is that a large proportion of patients with long-term conditions, especially those in more deprived geographical areas, either do not have access to the internet or do not perceive themselves to have the necessary technological skills required for computer-based telehealth interventions. Telehealth interventions may be acceptable to more people if access is facilitated as part of any intervention, particularly for patients in areas of socio-economic deprivation. The implication for routine health care provision is that at present telehealth should not be the only care option because there is a significant proportion of the population without sufficient technology skills or access to use it. For example, patients with depression would need to be offered cognitive behavioural therapy in forms other than computer-based ones (such as books) or offered considerable technological support.

Another important implication relates to the contextual issues of patients’ lives, with patients choosing not to participate because of health or domestic issues. Given this, triallists could aim to minimise the commitment and burden to participants of both the intervention and the trial. Additionally, there were some differences in the reasons for declining between the depression and CVD risk trials, which indicates that whilst there are similarities in telehealth trials, the specific reasons may differ depending on the population from which the trial is recruiting. It is recommended that future telehealth trials include a similar process of exploring why patients decline to participate to help build a picture of issues affecting participation for different health conditions.

Finally, a key reason for concern about low participation rates in trials is that this may lead to recruitment bias, with those patients included in the trial not being representative of those for whom the intervention would be provided in routine clinical practice. This was not the most pressing issue here. Most patients declining did so because they felt that telehealth was not suitable for them rather than for reasons related to the research.

## Conclusions

In both trials, patients from deprived general practices were more likely to decline the study invite. Patients provided a range of reasons for declining to participate in a telehealth trial, and these reasons were generally consistent with the literature from the few other telehealth trials, as well as from trials more generally. Some reasons were specific to telehealth, such as not having internet access; others were generic to all studies, such as being too busy. However, some reasons were specific to individuals’ health needs, and so were different across the two groups of long-term conditions recruited to this study. The primary reason for declining was due to technology issues; this was the case for patients who were registered at general practices in deprived areas. This has implications for the feasibility of both telehealth trials and telehealth in routine practice. For some patients, it was not clear if they were declining to participate in the trial or the intervention per se, for example, patients who said that they were not interested. If the latter, it raises questions about the acceptability of the telehealth intervention. It is recommended that other trials also explore why patients are not participating to facilitate a greater understanding of non-participation.

## Abbreviations

BME: Black and minority ethnic
CVD: Cardiovascular disease
RCT: Randomised controlled trial
